# Cell Wall Microdomains Analysis in the Quadrifids of *Utricularia dichotoma*

**DOI:** 10.3390/ijms26020832

**Published:** 2025-01-20

**Authors:** Bartosz J. Płachno, Małgorzata Kapusta, Marcin Feldo, Piotr Świątek

**Affiliations:** 1 Department of Plant Cytology and Embryology, Institute of Botany, Faculty of Biology, Jagiellonian University in Kraków, 9 Gronostajowa St., 30-387 Kraków, Poland; 2 Bioimaging Laboratory, Faculty of Biology, University of Gdańsk, 59 Wita Stwosza St., 80-308 Gdańsk, Poland; malgorzata.kapusta@ug.edu.pl; 3 Department of Vascular Surgery and Angiology, Medical University of Lublin, 16 Staszica St., 20-081 Lublin, Poland; martinf@interia.pl; 4 Institute of Biology, Biotechnology and Environmental Protection, Faculty of Natural Sciences, University of Silesia in Katowice, 9 Bankowa St., 40-007 Katowice, Poland; piotr.swiatek@us.edu.pl

**Keywords:** cell wall, cell wall microdomains, digestive trichomes, hemicelluloses, glands, Lentibulariaceae, scanning transmission electron microscopy, transfer cells, pectic homogalacturonan, xyloglucan, xylan

## Abstract

Carnivorous plants have fascinated botanists and ecologists with their various unusual adaptations in organ structure, physiology, and complex interactions with other organisms since the time of Charles Darwin. Species of the genus *Utricularia* (bladderworts, family Lentibulariaceae) are carnivorous plants that prey mainly on invertebrates using traps (bladders) of leaf origin. In the traps, there are glandular trichomes called quadrifids, which produce digestive enzymes and absorb the products of prey digestion. These quadrifids are unique due to their highly complex glandular cell structure; hence, they are an excellent model for studying the cell wall and its specialization. The main aim of the study was to investigate the presence and distribution of homogalacturonans (HGs) and hemicelluloses in the cell walls of trichome cells and especially in cell wall ingrowths in the quadrifid cells. The following antibodies were used against the wall components: anti-HGs (homogalacturonans) —JIM5 (low methylesterified HGs), JIM7 (highly esterified HGs), LM19 (low methylesterified HGs), CCRC-M38 (a fully de-esterified HG), LM5 (galactan); anti-hemicelluloses—LM25 (galactoxyloglucan; XXLLG, XXLG, XXXG modules of xyloglucans), LM15 (xyloglucan), CCRC-M138 (xylan), LM11 (heteroxylan); and anti-mannans: LM20 (heteromannan) and LM22 (heteromannan). The localization of the examined compounds was determined using immunohistochemistry techniques and immunogold labeling. In quadrifid cells, we found differences in the presence of the epitope detected by the LM5 antibody in the cell walls. In addition, cell wall ingrowths represented distinct microdomains of the cell wall in terms of the occurrence of wall components (they were methylesterified and demethylesterified homogalacturonan-poor). Hemicelluloses (galactoxyloglucan and xyloglucan) and arabinogalactans co-occur in cell wall ingrowths. Also, a part of the cell wall of the pedestal cell, which forms a Casparian strip, represented a distinct microdomain. We did not detect epitopes recognized by LM11, LM20 and LM22 antibodies. Our research shows that several cell wall microdomains occur in the cell walls of quadrifid cells. They differ depending on the presence and distribution of low methylesterified HGs, highly esterified HGs, fully de-esterified HGs, galactan (the epitope detected by the LM5 antibody), xyloglucan, galactoxyloglucan, and xylan (the epitope detected by the CCRC-M138 antibody).

## 1. Introduction

Carnivorous plants can lure, trap, and digest tiny organisms (protozoa, algae, animals) and utilize their nutrients. Carnivory has appeared many times in the evolution of flowering plants as an adaptation to poor habitats, which has resulted in many ways to capture prey [1,2,3]. Carnivorous plants have fascinated botanists and ecologists since the time of Charles Darwin and his book *Insectivorous Plants* [4]. Members of *Utricularia* L. (bladderworts) are carnivorous plants with very small but extremely fast-moving traps [5,6,7] for catching small animals and other organisms. These plants fascinate scientists for many reasons, and one is the unusual development and morphology (’fuzzy’ morphology) of the vegetative organs, which do not fit the classic division into root, leaf, and stem [8,9,10,11,12]. According to Rutishauser [13], bladderworts are the best-known examples of morphological misfits in vascular plants. This atypical construction plan already originates in embryonic development, which lacks an embryonic root and true cotyledons [14] and is associated with changes in the presence and expression of genes [15,16,17,18,19,20]. Also, a trend toward smaller genome sizes has been found among *Utricularia* [20,21,22]. Some changes in *Utricularia* genomes are probably connected to carnivory, as in other carnivorous plant genera, e.g., [23,24,25,26,27,28,29,30,31]. Whitewoods et al. [32] reported that simple shifts in gene expression are sufficient to form a trap in *Utricularia gibba*. Recently, Zedek et al. [33] detected significant correlations between *COX* mutations and smaller genome and chromosome sizes in Lentibulariaceae.

**Figure 1:**
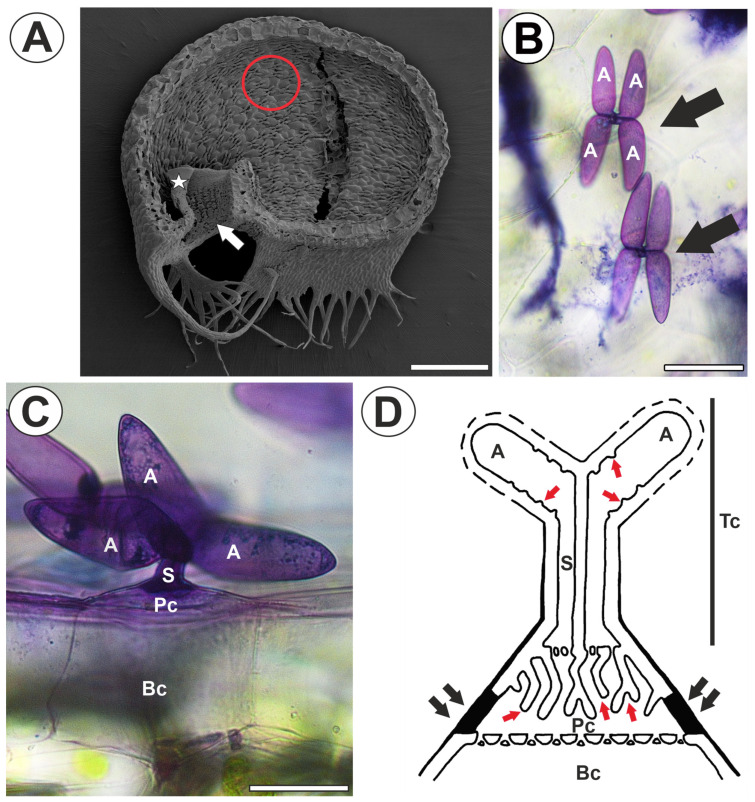
Trap structure of *Utricularia dichotoma* subsp. *novae-zelandiae*. Quadrifid structure. (**A**) A sagittally halved trap in scanning electron microscopy; trap door (star), trap entrance (arrow), with a red circle indicating the quadrifids, which are shown enlarged in (**B**), bar 500 µm. (**B**) The morphology of the quadrifids (arrow), treated with toluidine blue, arm (A), bar 20 µm. (**C**) Lateral view of the quadrifids, treated with toluidine blue; arm (A), stalk (s), pedestal cell (Pc), basal cell (Bc), bar 25 µm. (**D**) Diagram of quadrifid structure (after Fineran 1985 [40] modified); terminal cell (Tc), arm (A), stalk (s), pedestal cell (Pc), basal cell (Bc), cell wall ingrowths (red arrows), fully cutinized part of the cell wall of pedestal cell (black arrows), dashed line in arms indicates discontinuities in the cuticle.

The *Utricularia* traps are discoid, hollow bladders, usually 1–5 mm large. In many *Utricularia* species, the inner surface of the trap (except for the surface of the door and threshold) is covered with quadrifid hairs (=quadrifids), which have four terminal cells [34,35,36,37,38,39] (Figure 1A,B). The quadrifid is formed by basal, pedestal, and terminal cells (Figure 1C,D). These trichomes are highly specialized in the case of terminal cells. Each terminal cell consists of parts (a basal part, a stalk, and an arm) with distinct ultrastructures and functions [35,40]. The proximal regions of four terminal cells are united as a common stalk with a heavily impregnated, impervious outer wall with cutin. The protoplast of each cell in the stalk is narrow and has a longitudinally arranged tubular endoplasmic reticulum. At the distal end of the stalk, the cells separate first in opposite pairs, forming a cross piece, and then each pair’s cells separate in opposite directions to form the arms [35,40] (Figure 1B–D). There is also differentiation of the protoplast and cell wall in the arms. The nucleus and numerous mitochondria are concentrated towards the base of the arm. A large central vacuole occupies the middle and distal regions of the arm. Also, in this region, the cell wall forms small wall ingrowths [35,41,42] (Figure 1D). According to Fineran [40], this specialization of terminal cells is unique in the plant kingdom. This makes such a cell an extremely convenient object for studying cell wall microdomains [41,42]. Additionally, it is worth noting that the pedestal cell of this trichome is a transfer cell (sensu Pate and Gunning [43]) with an elaborate cell wall labyrinth [35,40,44]. Thus, quadrifids are a good model for transfer cell studies, especially since there are few models for detailed studies of the formation and structure of the wall labyrinth in flowering plants [45]. The main aim of this study was to investigate the presence and distribution of homogalacturonans (HGs) and hemicelluloses in the cell walls of trichome cells and especially in cell wall ingrowths in the quadrifid cells. We want to test the hypothesis of whether cell wall microdomains can be distinguished in the cell walls of quadrifids in terms of the occurrence of pectins and hemicelluloses. Previously, we studied the distribution of arabinogalactan proteins (AGPs) in quadrifid cells and found that AGPs did not occur randomly [41,42]. It is, therefore, intriguing to compare the presence of AGPs with other cell wall components using *Utricularia* trichomes as a model.

## 2. Results

### 2.1. Homogalacturonan Distribution

**Figure 2:**
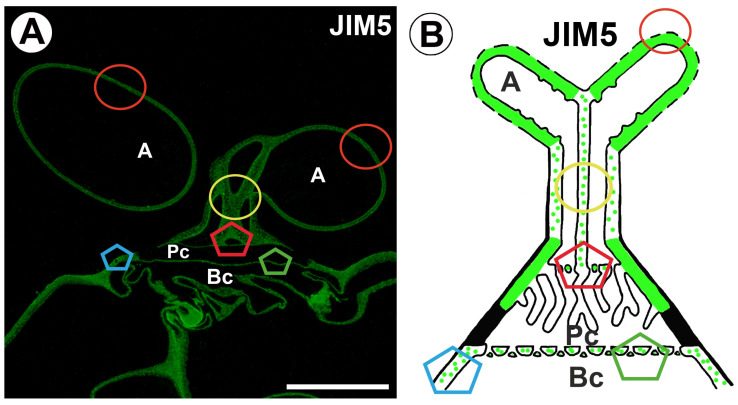
Labeling of cells with JIM5 (low methylesterified HG) in the quadrifid (green color—signal of antibody). The section positions are shown with circles and polygons on a schematic representation of quadrifids. (**A**) Section through quadrifid: arm (A), pedestal cell (Pc), basal cell (Bc), bar 10 µm and bar 10 µm. (**B**) Diagram of occurrence of low methylesterified HGs (detected by JIM5) in quadrifid cells; the arm of the terminal cell (A), pedestal cell (Pc), basal cell (Bc).

The epitope recognized by the JIM5 antibody (low methylesterified HGs) was detected in the cell walls of all quadrifid cell types: terminal, endodermal, and basal (Figure 2A,B). However, this epitope was missing from some parts of the cell walls. A fluorescence signal detected by JIM5 (low methylesterified HGs) was observed in the cell wall of the arm of the terminal cell (Figure 2A,B). In the proximal region of the arm of the terminal cell, the signal was absent from the cell wall ingrowths. In the stalk, in the common wall area between the protoplasts, this antibody signal displayed a discontinuous (dotted) pattern (Figure 2A and Figure S1).

**Figure 3:**
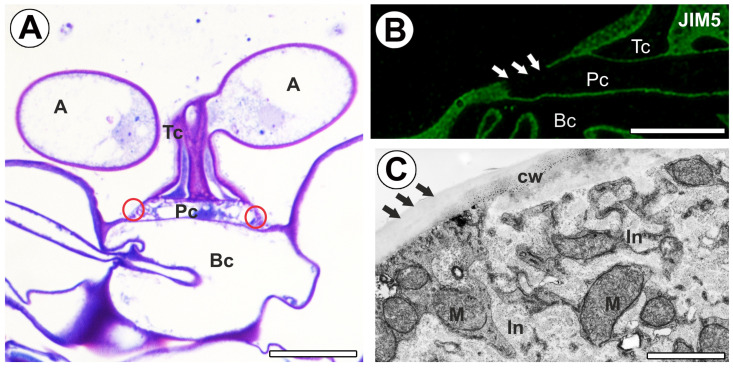
Labeling of cells with JIM5 (low methylesterified HG) in the quadrifid (green color—signal of antibody). (**A**) Semi-thin section through quadrifid, arm (A), terminal cell (Tc), pedestal cell (Pc), fully cutinized part of the cell wall of pedestal cell (indicated with a red circle), basal cell (Bc), bar 10 µm. (**B**) Section through quadrifid (magnification of Figure 2A), terminal cell (Tc), pedestal cell (Pc), basal cell (Bc); note no positive signal in the fully cutinized part of the cell wall of pedestal cell (white arrows); bar 5 µm. (**C**) Part of the pedestal cell seen in scanning transmission electron microscopy, fully cutinized part of the cell wall of pedestal cell (black arrows), cell wall ingrowths (In), mitochondrion (M), cell wall (cw), bar 1 µm.

A fluorescence signal occurred in the transverse wall between the terminal and pedestal cells (Figure 2A). A fluorescence signal occurred in the distal part of the lateral wall of the pedestal cell (Figure 2A,B). There was no fluorescence signal in part of the proximal region of the lateral wall of a pedestal cell. This part of the cell wall was entirely impregnated with cutin (Figure 3A,B). There was no fluorescence signal in the cell wall ingrowths of the pedestal cell. Fluorescence occurred in the transverse wall between the pedestal and basal cells (Figure 3B).

**Figure 4:**
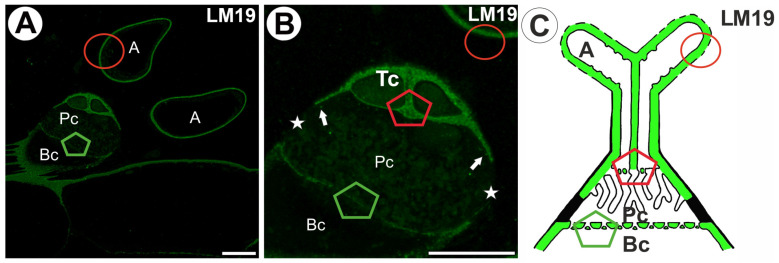
Labeling of cells with LM19 (low methylesterified HG) in the quadrifids (green color—a signal of antibody). The section positions are shown with circles and polygons on a schematic representation of quadrifids. (**A**,**B**) Section through quadrifid labeled with LM19, arm (A), terminal cell (Tc), pedestal cell (Pc), fully cutinized part of cell wall of pedestal cell (star), basal cell (Bc), wall ingrowths with positive signal (arrow); note part of pedestal cell without labeling (star); bar 10 µm and bar 10 µm. (**C**) Diagram of occurrence of low methylesterified HGs (detected by LM19) in quadrifid cells: the arm of the terminal cell (A), pedestal cell (Pc), basal cell (Bc).

A fluorescence signal detected by LM19 (low methylesterified HGs) was observed in the terminal cell (arm, stalk) (Figure 4A–C). This signal displayed a dotted pattern and occurred in the cell wall of a pedestal cell, except in the proximal region of the lateral wall of a pedestal cell (Figure 4A,B). A positive signal was observed in a few wall ingrowths in the pedestal cell’s lateral part (Figure 4B). The fluorescence signal detected by LM19 occurred in the cell walls of the basal cell (Figure 4B,C).

**Figure 5:**
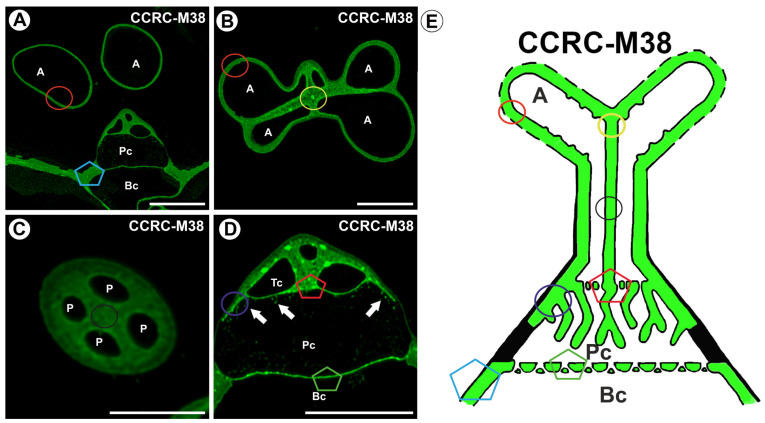
Labeling of cells with CCRC-M38 (low methylesterified HG) in the quadrifids (green color—a signal of antibody). The section positions are shown with circles and polygons on a schematic representation of quadrifids. (**A**) Section through quadrifid labeled with CCRC-M38, arm (A), pedestal cell (Pc), basal cell (Bc), bar 10 µm. (**B**) Section through arms (A) labeled with CCRC-M38, bar 10 µm. (**C**) Section through the stalk of quadrifid, labeled with CCRC-M38, note four protoplasts (P), bar 10 µm. (**D**) Section through quadrifid labeled with CCRC-M38, terminal cell (TC), pedestal cell (Pc), basal cell (Bc), wall ingrowths with positive signal (arrow), bar 10 µm. (**E**) Diagram of occurrence of low methylesterified HGs (detected by CCRC-M38) in quadrifid cells: the arm of the terminal cell (A), pedestal cell (Pc), basal cell (Bc).

A fluorescence signal detected by CCRC-M38 (a fully de-esterified HG) was observed in the terminal cell (arm, stalk) (Figure 5A–E). In the stalk and quadrifid capital, in the common wall area between the protoplasts, this antibody signal displayed a discontinuous (dotted) pattern (Figure 5A–E). A fluorescence signal occurred in the cell wall of the pedestal cell, except for the proximal region of the lateral wall of the pedestal cell (Figure 5E). A positive signal was observed in some wall ingrowths (Figure 5D). A fluorescence signal detected by CCRC-M38 occurred in the cell walls of the basal cell (Figure 5D,E).

**Figure 6:**
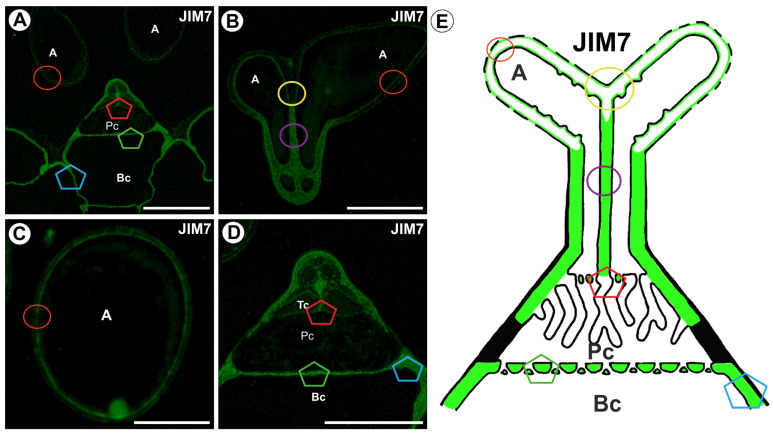
Labeling of cells with JIM7 (methylesterified HG) in the quadrifid (green color—signal of antibody). The section positions are shown with circles and polygons on a schematic representation of quadrifids. (**A**) Section through quadrifid, arm (A), pedestal cell (Pc), basal cell (Bc), bar 10 µm. (**B**) Section through arms (A), bar 10 µm. (**C**) Section through the arm (A), bar 10 µm. (**D**) Section through quadrifid, terminal cell (Tc), pedestal cell (Pc), basal cell (Bc) (magnification of Figure 6A), bar 10 µm. (**E**) Diagram of occurrence of methylesterified HGs (detected by JIM7) in quadrifid cells: the arm of the terminal cell (A), pedestal cell (Pc), basal cell (Bc).

A fluorescence signal from highly esterified HGs (detected by JIM7) was observed in the cell walls of the arms. The epitope was localized in the plasma membrane-proximal wall domain and the outer part of the cell wall (Figure 6A–E). An intense fluorescence signal occurred in the cell wall in the stalk. A fluorescence signal occurred in the cell wall of the pedestal cell, except in the proximal region of the lateral wall of a pedestal cell (Figure 6D,E). This was consistent with the observation of the presence of gold particles. A positive fluorescence signal was not observed in wall ingrowths (Figure 6D). A fluorescence signal detected by JIM7 occurred in the cell walls of the basal cell (Figure 6D,E).

**Figure 7:**
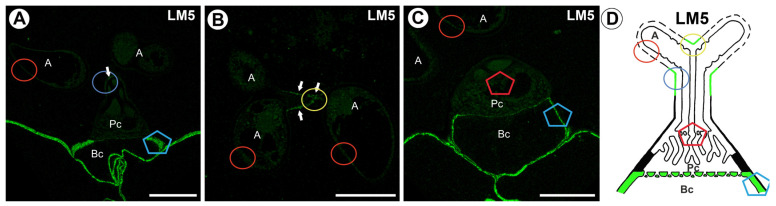
Labeling of cells with LM5 (galactan) in the quadrifid (green color—signal of antibody). The section positions are shown with circles and polygons on a schematic representation of quadrifids. (**A**) Section through quadrifid, arm (A), pedestal cell (Pc), basal cell (Bc), positive signal in stalk indicated by arrow, bar 10 µm. (**B**) Section through arms (A), positive signal indicated by arrow, bar 10 µm. (**C**) Section through quadrifid, arm (A), pedestal cell (Pc), basal cell (Bc), bar 10 µm. (**D**) Diagram showing the occurrence of galactan (detected by LM5) in quadrifid cells: arm of terminal cell (A), pedestal cell (Pc), basal cell (Bc).

The signal from the pectic polysaccharide (1–4)-β-D-galactan (detected by LM5) was observed in quadrifid only in the walls of the basal cell and outer cell wall in the stalk part of terminal cells (Figure 7A–D). No signal was observed in the pedestal cell. Also, no signal was observed in the wall ingrowths (Figure 7C,D).

### 2.2. Hemicellulose Distribution

**Figure 8:**
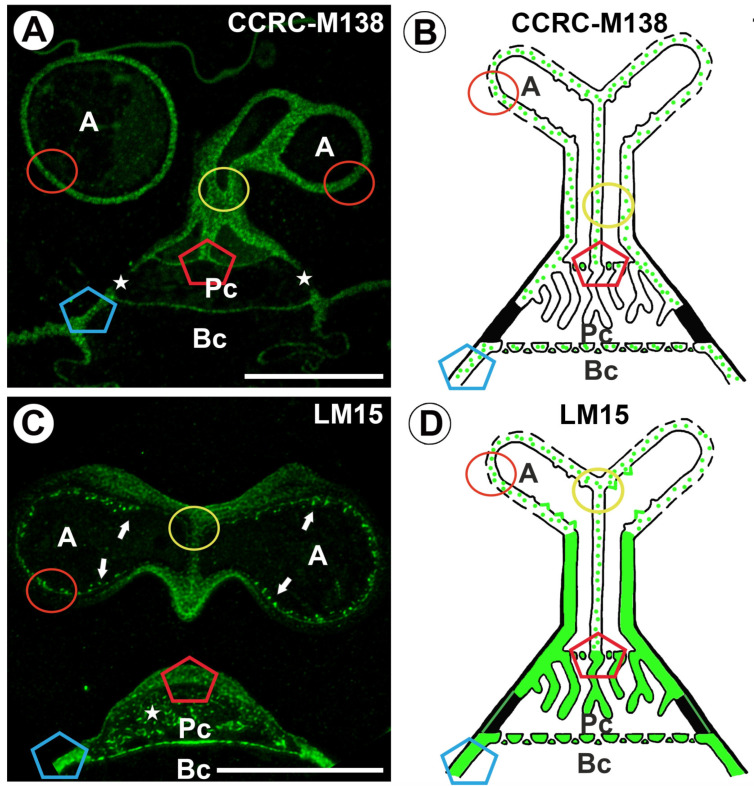
Labeling of cells with CCRC-M138 (xylan) and LM15 (xyloglucan) in the quadrifids (green color—a signal of antibody). The section positions are shown with circles and polygons on a schematic representation of quadrifids. (**A**) Section through quadrifid labeled with CCRC-M138, arm (A), pedestal cell (Pc), basal cell (Bc), note part of pedestal cell without labeling (star), bar 10 µm. (**B**) Diagram showing the occurrence of xylan (detected by CCRC-M138) in quadrifid cells: arm of terminal cell (A), pedestal cell (Pc), basal cell (Bc). (**C**) Section through quadrifid labeled with LM15, arm (A), pedestal cell (Pc), basal cell (Bc), positive signal in cell wall ingrowths in arms (arrow), note positive signal in cell wall ingrowths in pedestal cell (star), bar 10 µm. (**D**) Diagram showing the occurrence of xyloglucan (detected by LM15) in quadrifid cells: arm of terminal cell (A), pedestal cell (Pc), basal cell (Bc).

**Figure 9:**
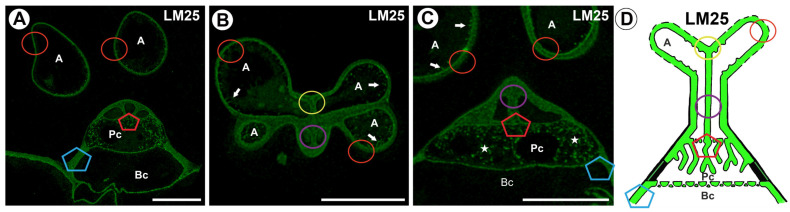
Labeling of cells with LM25 (galactoxyloglucans) in the quadrifids (green color—a signal of antibody). The section positions are shown with circles and polygons on a schematic representation of quadrifids. (**A**) Section through quadrifid, arm (A), pedestal cell (Pc), basal cell (Bc), bar 10 µm. (**B**) Section through arm of terminal cell (A), positive signal in cell wall ingrowths in arms (arrow), bar 10 µm (**C**) Section through quadrifid labeled, arm (A), pedestal cell (Pc), basal cell (Bc), positive signal in cell wall ingrowths in arms (arrow), note positive signal in cell wall ingrowths in pedestal cell (star), bar 10 µm. (**D**) Diagram showing the occurrence of galactoxyloglucans (detected by LM25) in quadrifid cells: arm of terminal cell (A), pedestal cell (Pc), basal cell (Bc).

A fluorescence signal detected by CCRC-M138 (which recognizes the glycan group of Xylan-6) was observed in the terminal cell (arm, stalk) (Figure 8A,B). The antibody signal displayed a discontinuous (dotted) pattern (Figure 8A,B). No positive signal was observed in wall ingrowths of terminal cells (Figure 8A,B). A fluorescence signal occurred in the cell wall of the pedestal cell, except in the proximal region of the lateral wall of the pedestal cell (Figure 8A,B). No positive signal was observed in wall ingrowths (Figure 9B). A fluorescence signal detected by CCRC-M138 occurred in the cell walls of the basal cell (Figure 8A,B).

The epitope recognized by the LM15 antibody (which reacts with the XXXG motif of land plants xyloglucan) was detected in the cell walls of all quadrifid cell types: terminal cell, endodermal cell, and basal cell (Figure 8C,D). The antibody signal displayed a discontinuous (dotted) pattern (Figure 8C,D). A fluorescence signal detected by LM15 was observed in the cell wall of the arm of the terminal cell and cell wall ingrowths (Figure 8C). In the stalk, there was a strong antibody signal in the outermost region in the wall of the stalk (Figure 8C,D). A fluorescence signal occurred in the cell wall of a pedestal cell and the proximal region of the lateral wall of the pedestal cell (Figure 8C,D). A positive signal was observed in the wall ingrowths of the pedestal cell (Figure 8C,D).

The epitope recognized by the LM25 antibody (which recognizes land plants galactoxyloglucans) was detected in the cell walls of all quadrifid cell types: terminal, endodermal, and basal (Figure 9A–D). The signal of the antibody displayed a discontinuous (dotted) pattern. A fluorescence signal detected by LM25 was observed in the cell wall of the arm of the terminal cell and cell wall ingrowths (Figure 9B). In the stalk, there was a strong antibody signal in the outermost region in the wall of the stalk (Figure 9C). A fluorescence signal occurred in the cell wall of the pedestal cell and the proximal region of the lateral wall of the pedestal cell (Figure 9C,D). A positive signal was observed in the wall ingrowths of the pedestal cell (Figure 9C,D).

**Figure 10:**
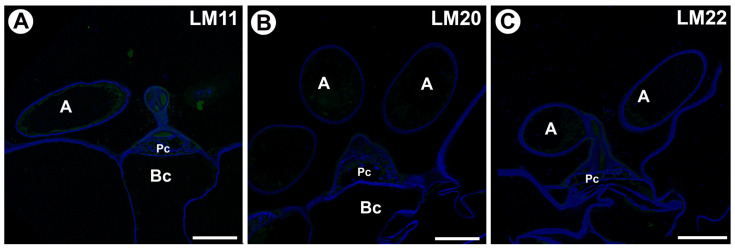
Labeling of cells with LM11 (heteroxylan), LM20 (heteromannan) and LM22 (heteromannan) in the quadrifids (green color —a signal of antibody, blue color—cellulose stained by Calcofluor White), arm (A), pedestal cell (Pc), basal cell (Bc). (**A**) Section through quadrifid labeled with LM11, bar 10 µm. (**B**) Section through quadrifid labeled with LM20, bar 10 µm. (**C**) Section through quadrifid labeled with LM22, bar 10 µm.

We did not find an epitope recognized by the LM211 (heteroxylan) antibody in the cell walls of quadrifids (Figure 10A).

### 2.3. Mannan Distribution

We did not find epitopes recognized by the LM20 (heteromannan) (Figure 10B) and LM22 (heteromannan) (Figure 10C) antibodies in the cell walls of quadrifids.

## 3. Discussion

Pectins are an essential polysaccharide component of the plant cell wall; they occur predominantly in the primary wall and the middle lamella and strongly affect its properties, e.g., cell wall extensibility and porosity. They play a role in many processes, e.g., cell-cell adhesion, plant growth, morphogenesis, organogenesis, defense, leaf abscission, and plant reproduction. Pectin can be divided into five main groups: apiogalacturonan, homogalacturonan (HG), xylogalacturonan, and rhamnogalacturonan I and rhamnogalacturonan II [46,47,48,49,50,51]. Plants use methyl-esterification and dimethyl-esterification of homogalacturonans for regulatory processes and development [49,52,53,54,55,56]. Cell walls of trap cells of *Utricularia dichotoma* subsp. *novae-zelandiae* contain HGs. Also, in other carnivorous plant traps, epidermal cells and parenchyma cell walls in *Aldrovanda vesiculosa* and *Dionaea muscipula* are rich in HGs [57,58]. Płachno et al. [57] suggested that this is related to the origin of the traps as transformed leaves.

We found that methylesterified and demethylesterified HG epitopes in *Utricularia* were abundant in the cell walls of quadrifid cells (in basal, pedestal, and terminal cells). However, in bifid trichomes and digestive glands from *Aldrovanda vesiculosa* traps [57,59] and in stellate trichomes in *D. muscipula* [58], terminal cells were poor in both low and highly esterified HGs. It was found that HGs may enhance cell wall strength [60]. One of the reasons is that the demethylesterified HGs can form egg boxes with calcium ions, thus strengthening cell walls. Thus, the occurrence of HGs in the cell walls of the pedestal cell and the cell wall of the stalk may be related to the mechanical role of these parts of the quadrifid, which have to support the expanded parts of the trichome (large arms). However, we did not find homogalacturonans in cell wall ingrowths in terminal cells of quadrifids; therefore, pectins represent a separate domain from the cell wall of the terminal cell. A more complicated picture emerges from analyzing cell wall ingrowths in the pedestal cell. We did not detect highly esterified HGs in these cell wall ingrowths (labeled by JIM7). Also, we did not detect demethylesterified HGs in these cell wall ingrowths (labeled by JIM5). However, demethylesterified HG epitopes (labeled by CCRC-M38 and LM19) occurred in only some cell wall ingrowths. This indicates differences in the structure of cell wall ingrowths, even within a single cell. The methylesterified HGs were not found in the wall ingrowths in gland cells of other carnivorous plant species: Droseraceae: *Aldrovanda vesiculosa* [57,59], *D. muscipula* [58], and Drosophyllaceae: *Drosophyllum lusitanicum* [61]. However, methylesterified HGs were found in the wall ingrowths of different plant species in other flowering plants [62] and bryophytes [63,64].

It is exciting to observe the lack of antibody labeling (except for hemicelluloses detected by LM15 [xyloglucan] and LM25 [galactoxyloglucans]) in the proximal region of the lateral wall of a pedestal cell of a quadrifid. This part of the cell wall is cutinized and acts as a Casparian strip, as Fineran and Gilbertson [65] demonstrated using lanthanum nitrate and uranyl acetate salts as tracers. On the other hand, it is still unknown to what extent this part of the wall is different in composition or whether this is due to the availability of cell wall components for antibodies. On the other hand, substantial cell wall modification, e.g., lignification, allows the detection of wall components by antibodies [66,67]. For example, in *Utricularia neottioides*, an extensin epitope (JIM11) was detected in sclerenchyma fibers and vessel cell walls [68]. Perhaps treatment with the appropriate enzymes (cutinase) could expose sites for antibodies, but it is an open question whether such interference would destroy the composition of the cell wall.

β-1,4-Galactan plays a vital role in modulating the mechanical properties of the cell wall [69,70], as was confirmed using the *Arabidopsis thaliana* mutant [71]. Therefore, it is not surprising that β-1,4-galactan was found in cell walls in tissues responsible for mechanical resistance of organs, e.g., phloem fibers and secondary xylem cells in the stem of *Populus trichocarpa* [67] and in phloem and xylem in roots of *Ceratopteris richardii* and *Arabidopsis thaliana* [72]. β-1,4-Galactan was also observed in water-conducting cells in some mosses and liverworts [73,74]. Moreover, β-1,4-galactan may be accumulated in the cell walls under stress. It was found that salt stress induced the accumulation of β-1,4-galactan in the root cell walls of *Arabidopsis thaliana* [75]. Also, in *A. thaliana*, it was demonstrated that β-1,4-galactan has a role in cell growth and elongation [76]. β-1,4-Galactan is a water-retaining viscoelastic component of the cell wall. Thus, it increases cell wall flexibility and has a role in cell shape [77], which explains the presence in the walls of the phloem cells (in sieve tubes), which are exposed to turgor-related deformations [72]. Here, we found pectic polysaccharide β-1,4-galactan (detected by LM5) in the basal cell of the quadrifid (showing an intense signal), which brings these cells closer to the epidermal trap cells in comparison with the pedestal cell and terminal cells. β-1,4-Galactan was also present in the stalk (only in the superficial part of the cell walls), which may be related to the mechanical stress to which this part of the quadrifid is exposed. We did not detect β-1,4-galactan in cell wall ingrowths in either the terminal or pedestal cells of the quadrifid. It should be noted that further studies using more specific antibodies will determine whether there is, in fact, a lack of galactan side chains or there is no LM5 binding because there is no rhamnogalacturonan I in the cell walls of pedestal and terminal cells.

Also, galactan (or, more precisely, the epitope recognized by the LM5 antibody) was not found in the cell wall ingrowths of *Aldrovanda vesiculosa* cells of digestive glands [59] or in bifid trichomes of this species [57] or in stellate trichomes in *Dionaea muscipula* [58]. Moreover, Ligrone et al. [78] did not detect galactan in the cell wall ingrowths in transfer cells of *Elodea canadensis* leaves. However, this compound was found in the wall ingrowths in transfer cells of *Vicia faba* only in the inner region of wall ingrowths [62]. Galactan also occurs in cell wall ingrowths in some bryophytes [63,79].

Hemicelluloses are polysaccharides essential for strengthening the cell wall by interacting with cellulose [80]. They also regulate cell wall expansibility and cell-to-cell adhesion [81,82]. Hemicelluloses comprise diverse polymers, including xylans, xyloglucans, mannans, and mixed-linkage glucans [83]. Xylan interacts with cellulose and other hemicelluloses. The secondary cell wall regulates the interface between cellulose and lignin [83,84]. We found that the xylan recognized by the CCRC-M138 antibody was present in the cell walls of quadrifid cells. However, it was lacking in cell wall ingrowths. However, this result does not mean there is no xylan, but only that the epitope detected by the CCRC-M138 antibody is absent.

Our study demonstrated that the hemicelluloses recognized by the LM25 (for galactoxyloglucan) and LM15 antibodies (for xyloglucan) were present in quadrifid cells. However, unlike xylan, these hemicelluloses occur in the cell wall ingrowths. It is exciting to note the occurrence of these hemicelluloses (galactoxyloglucan and xyloglucan) and AGPs (recognized by the JIM13 and JIM8 antibodies) in cell wall ingrowths [42]. This pattern of co-occurrence of hemicelluloses with AGPs is also found in cell wall ingrowths in the cells of trichomes and glands of other carnivorous plants: Droseraceae: *Aldrovanda vesiculosa* [57,59], *D. muscipula* [58], and Drosophyllaceae: *Drosophyllum lusitanicum* [61]. Some hemicelluloses were also localized in cell wall ingrowths in epidermal transfer cells of *Vicia faba* cotyledons [62] as well as in cells of the bryophyte *Physcomitrium patens* [63]. Since some hemicelluloses participate in cell wall expansion and regulator of cell wall extensibility [85,86], this can explain their prevalence in cell wall ingrowths in different species. We found no epitopes recognized by the LM11 (heteroxylan), LM20 (heteromannan) and LM22 (heteromannan) antibodies in the cell walls of quadrifids. In the future, it should be determined whether they are absent or present in cell walls in other organs of this species.

**Figure 11:**
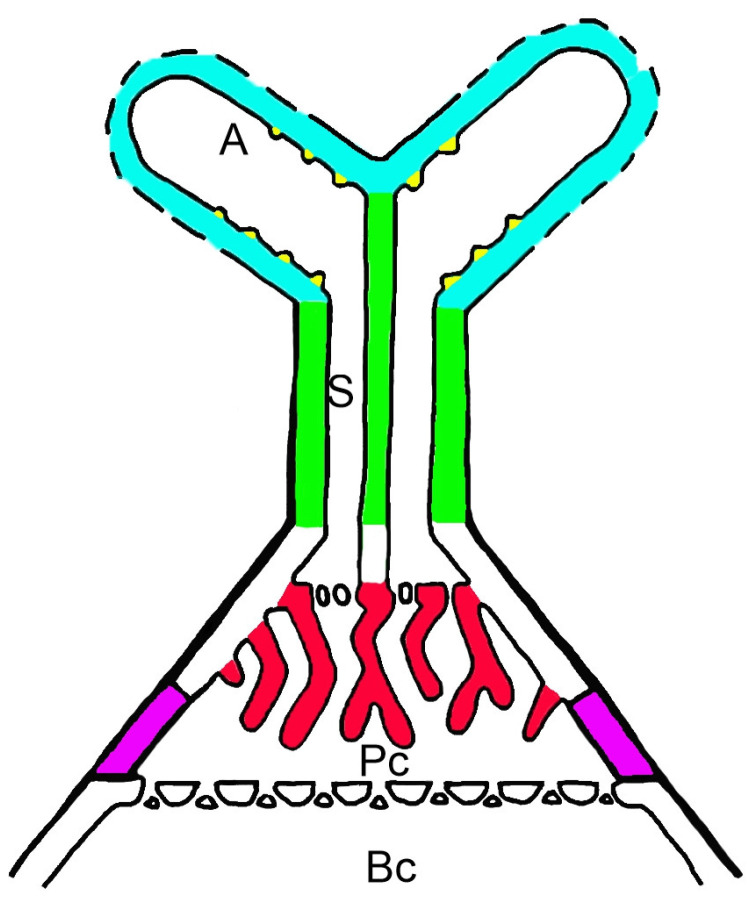
A model depicting cell wall microdomains of quadrifids; Cell wall microdomains: cell wall ingrowths of the pedestal cell (red color), cell wall ingrowths of the terminal cell (yellow color), the cell wall of the stalk of the terminal cell (green color), the cell wall of the arm (blue color), and part of the cell wall of the pedestal cell (pink); basal cell (Bc), pedestal cell (Pc), stalk (S), arm (A).

Recently, Dauphin et al. [87] defined the term “cell wall microdomains” and described different types of them. They stated that a cell wall microdomain corresponds to a wall territory with a specific molecular composition, some of which could correspond to specific molecular domains. According to this definition, several cell wall microdomains occur in quadrifid cells (Figure 11). They differ depending on the presence and distribution of low methylesterified HGs, highly esterified HGs, fully de-esterified HGs, galactan (the epitope detected by the LM5 antibody), xyloglucan, galactoxyloglucan, and xylan (the epitope detected by the CCRC-M138 antibody): cell wall ingrowths of the pedestal cell, cell wall ingrowths of the terminal cell, the cell wall of the stalk of the terminal cell, the cell wall of the arm, and part of the cell wall of the pedestal cell (which is highly cutinized) (Figure 11). Similar cell wall microdomains have been shown in cells of the outer glands of *Utricularia* [88].

## 4. Materials and Methods

### 4.1. Plant Material

*Utricularia dichotoma* subsp. *novae-zelandiae* (Hook. f) R.W.Jobson [89] plants were grown in the greenhouses of the Botanical Garden of the Jagiellonian University. The plants were cultivated in wet peat under natural sunlight exposure. Ten traps were analyzed. Traps were randomly selected for analysis.

### 4.2. Histological and Immunochemical Analysis

The traps were fixed in 8% (*w*/*v*) formaldehyde (PFA, Sigma-Aldrich, Sigma-Aldrich Sp. z o.o. Poznań, Poland) mixed with 0.25% (*v*/*v*) glutaraldehyde (GA, Sigma-Aldrich, Sigma-Aldrich Sp. z o.o. Poznań, Poland) in a PIPES buffer overnight at 4 °C. The PIPES buffer contained 50 mM PIPES (piperazine-N,N′-bis [2-ethanesulfonic acid], Sigma-Aldrich, Sigma-Aldrich Sp. z o.o. Poznań, Poland), 10 mM EGTA (ethylene glycol-bis[β-aminoethyl ether]N,N,N′,N′-tetraacetic acid, Sigma Aldrich, Poznań, Poland), and 1 mM MgCl_2_ (Sigma-Aldrich, Sigma-Aldrich Sp. z o.o. Poznań, Poland), pH 6.8. For analysis of the occurrence of the major cell wall polysaccharides and glycoproteins, the plant material was dehydrated with acetone and embedded in an Epoxy Embedding Medium Kit (Fluka). Ultrathin sections were cut on a Leica Ultracut UCT ultramicrotome. The rehydrated sections in PBS buffer were blocked with 1% bovine serum albumin (BSA, Sigma-Aldrich) in a PBS buffer and incubated with the following primary antibodies overnight at 4 °C (Table 1): anti-homogalacturonans (HG) and anti-hemicelluloses [66,90,91,92,93,94,95,96,97,98]. All of the primary antibodies were used in a 1:20 dilution. They were purchased from Plant Probes, UK (rat monoclonal antibodies: JIM5, JIM7, LM19, LM25, and LM15) and Agrisera, Sweden (mouse monoclonal antibodies: CCRC-M38, and CCRC-M138).

Secondary antibodies—goat anti-rat secondary or anti-mouse antibody conjugated with FITC or Alexa Fluor 488, respectively—were purchased from Abcam (Cambridge, UK). The samples were then cover-slipped using a Mowiol mounting medium: a mixture of Mowiol 4-88 (Sigma-Aldrich) and glycerol for fluorescence microscopy (Merck, Warsaw, Poland) with the addition of 2.5% DABCO (Carl Roth GmbH + Co. KG, Karlsruhe, Germany). They were viewed using a Leica STELLARIS 5 WLL confocal microscope with Lightning deconvolution. For each trap analyzed, at least two replicates were made for each antibody used. Negative controls were created by omitting the primary antibody step, which caused no fluorescence signal in any of the control frames for any stained slides (Figure S2). Crystalline cellulose was also labeled using Calcofluor White Stain (Merck Life Science Sp. z o.o., an affiliate of Merck KGaA, Darmstadt, Germany). Semi-thin sections (0.9–1.0 µm thick) were prepared for light microscopy (LM) and stained for general histology using aqueous methylene blue/azure II (MB/AII) for 1–2 min.

In addition, live traps were sectioned and treated with toluidine blue (Sigma-Aldrich, Sigma-Aldrich Sp. z o.o., Poznań, Poland).

### 4.3. Scanning Transmission Electron Microscopy

The glands were also examined using electron microscopy, as follows: Fragments of the traps were fixed in a mixture of 2.5% glutaraldehyde with 2.5% formaldehyde in a 0.05 M cacodylate buffer (Sigma-Aldrich, Sigma-Aldrich Sp. z o.o., Poznań, Poland; pH 7.2) a few days, and later the material was processed as described by Płachno et al. [99]. Ultrathin sections were cut on a Leica Ultracut UCT ultramicrotome. The sections were examined using a Hitachi UHR FE-SEM SU 8010 (Hitachi, Tokyo, Japan) microscope equipped with a transmitted electron detector housed at the University of Silesia in Katowice.

## 5. Conclusions

Our cytological study showed the presence of various cell wall components in the cell walls of quadrifid cells: methylesterified and demethylesterified homogalacturonans, galactan, xylan, galactoxyloglucan, and xyloglucan. The co-occurrence of hemicelluloses (galactoxyloglucan and xyloglucan) with AGPs in the ingrowths of cell walls is of interest.

We found differences between cells in the occurrence of wall components, primarily in the presence of epitopes detected by the LM5 antibody. We also saw the following cell wall microdomains. The absence of methylesterified and demethylesterified homogalacturonans and the epitope detected by the CCRC-M138 antibody distinguished cell wall ingrowths in terminal cells. The absence of xylan, the epitope detected by the CCRC-M138 antibody, and methylesterified homogalacturonans distinguished cell wall ingrowths in the pedestal cell. These ingrowths either lacked demethylesterified homogalacturonans, or only these homogalacturonans were restricted to certain ingrowths. A particularly noteworthy part of the cell wall functions as a Casparian strip in the pedestal cell. Here, we found no antibody signal (except for galactoxyloglucan detected by LM15 and xyloglucan detected by LM25), possibly due to the cell wall modification. Future research should focus on the relationship between cell wall modification and antibody epitope accessibility. Our research contributes to a better description of the cell wall microdomains of quadrifids. However, by using more specific antibodies, e.g., LM28 (which recognizes land plants glucuronoxylan) [100,101] and an RG-I backbone-specific antibody, it will be possible to find more microdomains in quadrifids.

## Figures and Tables

**Table 1:** Specificities of the monoclonal antibodies against different cell wall epitopes [66,90,91,92,93,94,95,96,97,98].

Cell Wall Polysaccharides	Monoclonal Antibody	Specificity
pectins	JIM5	low methylesterified HGs
	LM19	low methylesterified HGs
	JIM7	highly esterified HGs
	CCRC-M38	a fully de-esterified HG
	LM5	galactan
hemicelluloses	LM25	galactoxyloglucan (XLLG, XXLG, XXXG modules)
	LM15	xyloglucan (XXXG module)
	CCRC-M138	xylan
	LM11	heteroxylan, unsubstituted and relatively low-substituted xylans
	LM20	heteromannan
	LM22	heteromannan, glucomannan, β-(1→4)-manno-oligosaccharides from DP2 to DP5

## Data Availability

The data presented in this study are available on request from the corresponding author.

## Supplementary Materials

The supporting information can be downloaded at: https://www.mdpi.com/article/10.3390/ijms26020832/s1.
