# Supplementary material for: Cell Wall Microdomains Analysis in the Quadrifids of Utricularia dichotoma
Source: Int J Mol Sci. 2025 Jan 20;26(2):832. doi: 10.3390/ijms26020832 (PMC11766393; doi:10.3390/ijms26020832)

Figure S1

Figure S1. A-C. Labeling of cells with JIM5 (low methylesterified HG) in the quadrifid - different optical sections, bar 10 μm.

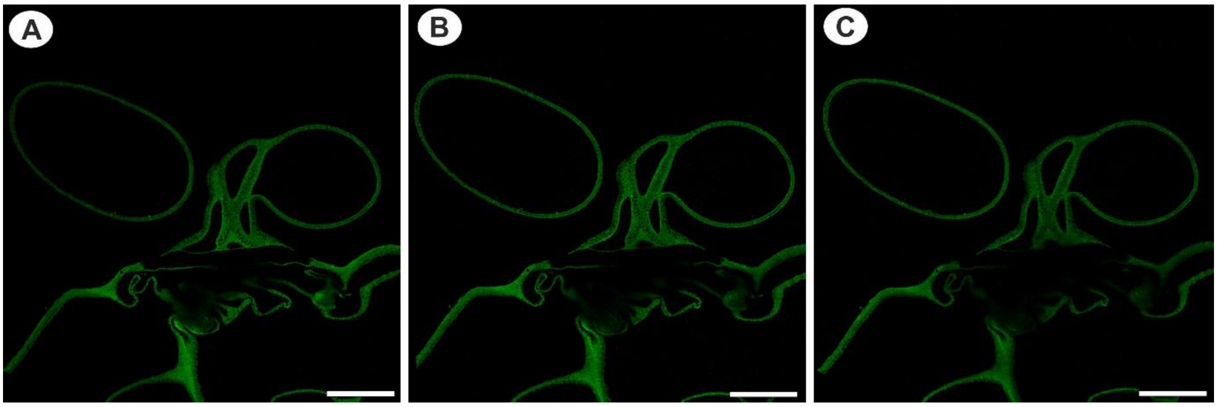

Figure S2

Figure S2. A-B. Control reactions of cell wall components after immunolabeling, section through the quadrifids, bar 10 μm.

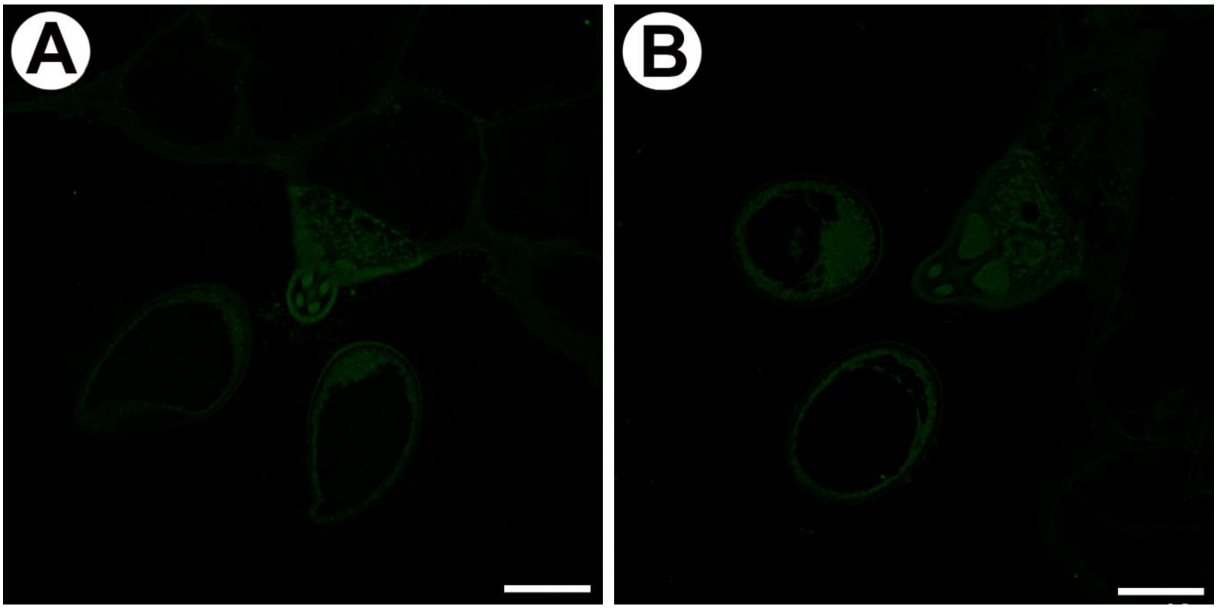

Supplement: Supplementary file 1 [file ijms-26-00832-s001.zip › ijms-3405015-supplementary.pdf]
